# Mutation Spectrum of *EGFR* From 21,324 Chinese Patients With Non-Small Cell Lung Cancer (NSCLC) Successfully Tested by Multiple Methods in a CAP-Accredited Laboratory

**DOI:** 10.3389/pore.2021.602726

**Published:** 2021-04-07

**Authors:** Linlin Mao, Weiwei Zhao, Xiaoxia Li, Shangfei Zhang, Changhong Zhou, Danyan Zhou, Xiaohua Ou, Yanyan Xu, Yuanxiao Tang, Xiaoyong Ou, Changming Hu, Xiangdong Ding, Pifu Luo, Shihui Yu

**Affiliations:** ^1^ Clinical Genome Center, KingMed Diagnostics, Guangzhou, China; ^2^ Institute of KingMed Translational Medicine, Guangzhou, China; ^3^ Department of Pathology, KingMed Diagnostics, Guangzhou, China

**Keywords:** non-small cell lung cancer, EGFR mutation testing, Sanger sequencing, real-time PCR, next-generation sequencing

## Abstract

Genotyping epidermal growth factor receptor (*EGFR*) gene in patients with advanced non-small cell lung cancers (NSCLC) is essential for identifying those patients who may benefit from targeted therapies. Systemically evaluating *EGFR* mutation detection rates of different methods currently used in clinical setting will provide valuable information to clinicians and laboratory scientists who take care of NSCLC patients. This study retrospectively reviewed the *EGFR* data obtained in our laboratory in last 10 years. A total of 21,324 NSCLC cases successfully underwent *EGFR* genotyping for clinical therapeutic purpose, including 5,244 cases tested by Sanger sequencing, 13,329 cases tested by real-time PCR, and 2,751 tested by next-generation sequencing (NGS). The average *EGFR* mutation rate was 45.1%, with 40.3% identified by Sanger sequencing, 46.5% by real-time PCR and 47.5% by NGS. Of these cases with *EGFR* mutations identified, 93.3% of them harbored a single *EGFR* mutation (92.1% with 19del or L858R, and 7.9% with uncommon mutations) and 6.7% harbored complex *EGFR* mutations. Of the 72 distinct *EGFR* variants identified in this study, 15 of them (single or complex *EGFR* mutations) were newly identified in NSCLC. For these cases with *EGFR* mutations tested by NGS, 65.3% of them also carried tumor-related variants in some non-*EGFR* genes and about one third of them were considered candidates of targeted drugs. NGS method showed advantages over Sanger sequencing and real-time PCR not only by providing the highest mutation detection rate of *EGFR* but also by identifying actionable non-*EGFR* mutations with targeted drugs in clinical setting.

## Introduction

Lung cancer is the leading cause of cancer-related mortality worldwide [1, 2]. Approximately 610,000 lung cancer-related deaths were reported in China in 2015 [3]. Non-small cell lung cancer (NSCLC) is the most common histological subtype of lung cancer, accounting for approximately 80–85% of the disease. Targeted therapy based on the identification of actionable genetic/genomic alterations in the disease has led to integration of molecular testing for planning treatment strategies for advanced NSCLC patients [4, 5].

Activating mutation in the epidermal growth factor receptor gene (*EGFR*) is the most frequent genetic alteration in NSCLC. *EGFR* mutations can be detected in 15% of adenocarcinoma subtype of Caucasian NSCLC but in 40–50% of same subtype of East Asian NSCLC. Exon-19 deletions (19del) and L858R substitution in exon-21 are the 2 classical *EGFR* mutations which could predict tumor responses to EGFR tyrosine kinase inhibitors (EGFR-TKIs) in NSCLC patients [6–10]. Other uncommon *EGFR* mutations have also been found to show sensitiveness (eg., Exon-19 insertions, p.L861Q in exon-21, p.G719X in exon-18, and p.S768I in exon-20) or resistance (eg., most exon-20 insertions) to EGFR-TKIs. T790M substitution in exon-20 is a well-known acquired mutation resistant to first or second generation EGFR-TKIs, but sensitive to third generation EGFR-TKIs. Currently, genotyping *EGFR* has been recommended by both laboratory and clinical guidelines as evidence-based standard care for advanced NSCLC patients [11–14].

Various technical platforms are clinically available for genotyping *EGFR*, including commonly used methods, such as Sanger sequencing, real-time PCR and NGS and occasionally used methods, such as denaturing high performance liquid chromatography (DHPLC) and Luminex liquid chip. Systemically comparing *EGFR* mutation detection rates detected by these methods routinely used in clinical setting is rarely reported [15, 16]. In this study, we retrospectively reviewed our successfully tested *EGFR* results for 21,324 unselected Chinese NSCLC patients whose specimens were performed in our laboratory, a College of American Pathologists (CAP)-certified reference laboratory providing *EGFR* mutation testing for NSCLC patients.

## Materials and Methods

### Patients

In total, 21,324 Formalin-Fixed and Paraffin-Embedded (FFPE) tumor specimens of NSCLC patients from 30 provinces of China were successfully performed in our laboratory for testing *EGFR* mutations from June 2009 to December 2018. The *EGFR* testing results were retrospectively reviewed and reported with exclusion of those cases with failed *EGFR* testing. For the cases with duplicated tests, only the result of the first successful test was counted. Ages of these patients ranged from 16 to 96 years old (median: 63 years old) and the gender percentages were 56.9% for females, 41.5% for males, and 1.6% with unknown gender information. All the *EGFR* tests were ordered by physicians for therapeutic purposes and were performed at a single testing center. This study was approved by the ethics review board of KingMed Diagnostics.

### Identification of Epidermal Growth Factor Receptor Mutations

The numbers of cases tested by different methods were shown in Supplementary Figure S1. According to the standard operation procedures (SOPs) validated in our laboratory, prior to *EGFR* mutation testing, tumor cell content (TCC) of the specimens was assessed by at least an experienced pathologist. A specimen with ≥20% TCC was required by Sanger sequencing, and recommended for real-time PCR and NGS methods. A specimen with 1–20% TCC was acceptable for testing *via* real-time PCR or NGS but not by Sanger sequencing. DNA from the specimens was extracted using QIAamp DNA FFPE Tissue Kit according to the manufacturer’s protocol (Qiagen China, Shanghai, China). MagMAX FFPE DNA/RNA Ultra Kit was used to isolate both DNA and RNA from the same section of FFPE tissues for the amplicon-based NGS testing (Life Technologies Corporation, Austin, United States). Technical parameters, such as sensitivities and specificities etc., were determined before these methods were used in clinical setting (data not shown).

Sanger sequencing for genotyping *EGFR* was launched since the year of 2009 and a total of 5,244 cases successfully tested by this technique was included in this study. *EGFR* exons-18 to -21 were amplified using polymerase chain reaction (PCR) and directly sequenced using ABI 3730xl (Applied Biosystems, Foster City, United States).

Since 2013, real-time PCR-based method for detecting *EGFR* mutations was applied in our laboratory using a commercial kit, *EGFR* RGQ PCR Kit (Qiagen China, Shanghai, China). A total of 13,329 cases successfully tested by this technique was included in this study. This technique covers 29 known mutations spanning exons-18 to -21 including exon-18 missense mutations at G719X (G719S, G719A and G719C), exon-19 deletions (19del), exon-20 missense mutations (S768I and T790M) and insertions (20ins), and exon-21 missense mutations (L858R and L861Q). Genotyping *EGFR* using NGS was launched in 2016 and so far, a total of 2,751 cases was successfully performed and the results were included in this study. For the first 1,089 cases, Ion AmpliSeq™ Colon and Lung Cancer Panel V2 and Ion AmpliSeq™ RNA Fusion panel were used covering point mutations and small insertions and deletions (indels) of 22 genes (*EGFR*, *ALK*, *BRAF*, *KRAS*, *MET*, *ERBB2*, *AKT1*, *CTNNB1*, *ERBB4*, *DDR2*, *FBXW7*, *FGFR1*, *FGFR2*, *FGFR3*, *MAP2K1*, *NOTCH1*, *NRAS*, *PIK3CA*, *PTEN*, *SMAD4*, *STK11* and *TP53*) as well as fusions of 4 genes (*ALK*, *ROS1*, *RET* and *NTRK1*) (ThermoFisher, Waltham, United States). For these cases, sequencing was performed on an Ion Torrent PGM instrument and data analysis was performed using Torrent Suite Software and Torrent Server. For the remaining 1,662 cases, we adopted a validated capture-based method for library preparation (Integrated DNA Technologies, Inc., Coralville, United States) and performed DNA sequencing using Illumina Nextseq 500 or NovaSeq 6000 systems (Illumina, San Diego, United States). After sequencing, a clinically validated bioinformatics pipeline was used to identify variants in the targeted genes. Sequence variants were interpreted and reported according to the guideline compiled by Association of Molecular Pathology (AMP) [17]. In brief, tier 1 (variants of strong clinical significance) and tier 2 (variants of potential clinical significance) variants were reported while tier 3 (variants of unknown clinical significance) and tier 4 (benign or likely benign variants) variants were not reported. Detailed technical procedures for all the 3 methods were listed in Supplementary Material.

### Statistical Analysis

We used Chi-square testing for comparing *EGFR* mutation detection rates identified by the 3 methods. *p*-values less than 0.05 were considered statistically significant in all scenarios. IBM SPSS Statistics Version 19 was used for all statistical analysis.

## Results

### Tumor Cell Content in Different Testing Groups

Our records showed that 5,244 NSCLC samples with 20–90% TCCwere successfully tested by Sanger sequencing, 13,329 NSCLC samples with 1–95% TCC were tested by real-time PCR, and 2,751 NSCLC samples with 1–90% TCC were tested by NGS. In details, 58.5% (12,484/21,324) of the samples were recorded with <40% TCC accounting for 46.8% (2,454/5,244) of the samples tested by Sanger sequencing, 60.8% (8,109/13,329) tested by real-time PCR, and 69.8% (1,921/2,751) tested by NGS; 20.4% (4,359/21,324) of the samples were recorded with <20% TCC accounting for 26.3% (3,509/13,329) tested by real-time PCR and 30.9% (850/2,751) tested by NGS; 2.3% (490/21,324) of the samples were recorded with 1% TCC accounting for 3.2% (429/13,329) tested by real-time PCR and 2.2% (61/2,751) tested by NGS (Table 1).

**TABLE 1:** Percentages of NSCLC specimens with different tumor cell content (TCC) tested by different platforms.

Specimens	Sanger sequencing^a^	Real-time PCR	NGS
TCC <40%	46.8% (2,454/5,244)	60.8% (8,109/13,329)	69.8% (1,921/2,751)
TCC <20%	/	26.3% (3,509/13,329)	30.9% (850/2,751)
TCC = 1%	/	3.2% (429/13,329)	2.2% (61/2,751)

^a^ NSCLC specimens with ≥20% TCC were required for testing by Sanger sequencing.

### Epidermal Growth Factor Receptor Mutation Rates

**FIGURE 1:**
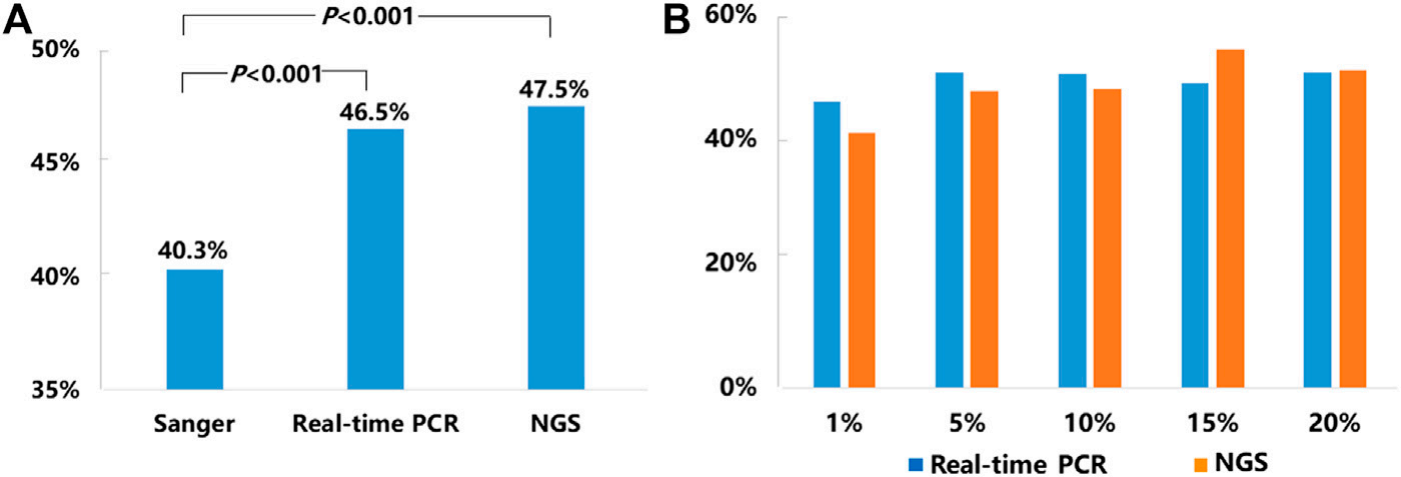
Mutation detection rates for *EGFR*. **(A)** The average mutation detection rates for *EGFR* obtained by Sanger sequencing, real-time PCR, and NGS methods. **(B)** *EGFR* mutation detection rates in NSCLC samples with 1–20% TCC by real-time PCR and NGS.

Of the 21,324 NSCLC samples successfully tested for *EGFR*, 9,621 of them carried somatic *EGFR* mutations, representing an average positive *EGFR* detection rate of 45.1% including 40.3% (2,111/5,244) tested by Sanger sequencing, 46.5% (6,202/13,329) tested by real-time PCR, and 47.5% (1,308/2,751) tested by NGS respectively (Figure 1A.). The detection rates obtained by real-time PCR and NGS methods were significantly higher than that obtained by Sanger sequencing (*p* < 0.001) (Figure 1A).

Since our SOPs allowed testing NSCLC samples with TCC from 1 to 20% by both real-time PCR and NGS methods, we compared *EGFR* mutation detection rates by these two methods in the samples with different levels of TCC (1, 5, 10, 15, and 20%). Detailed information was listed in Figure 1B. In brief, the positive detection rates of real-time PCR vs. NGS were 45.9% (197/429) vs. 41% (25/61) in 1% TCC, 50.7% (380/750) vs. 47.6% (79/166) in 5% TCC, 50.4% (765/1,518) vs. 48% (195/406) in 10% TCC, 49% (398/812) vs. 54.4% (118/217) in 15% TCC, and 50.6% (1,077/2,129) vs. 51.1% (258/505) in 20% TCC. Statistically, there were no significant difference about the detection rates by the 2 methods in each of the 5 subgroups (*p* > 0.05), although real-time PCR method showed higher detection rates in relatively lower TCC (1, 5, and 10%) in contrast to the higher detection rates in samples with relatively higher TCC (15 and 20%) by NGS.

Since NGS is a quantitative method for *EGFR* testing, we analyzed *EGFR* variant allele frequencies (VAFs) in the specimens with different TCC levels. The median *EGFR* VAFs were 0.074 (range: 0.01–0.675), 0.097 (range: 0.011–0.97), 0.161 (range: 0.01–0.902), 0.254 (range: 0.014–0.811), and 0.261 (range: 0.01–0.957) for NSCLC samples with 1, 5, 10, 15, and ≥20% TCC respectively. Although the median *EGFR* VAFs increase with increased TCC levels in the specimens tested, the values of *EGFR* VAFs overlapped greatly among groups with different TCC, showing very high *EGFR* VAF variations ranging from 0.01 to 0.97 (for example, *EGFR* VAF was up to 0.675 in one sample with 1% TCC, down to 0.01 in another sample with 20% TCC).

### Epidermal Growth Factor Receptor Mutation Patterns

**FIGURE 2:**
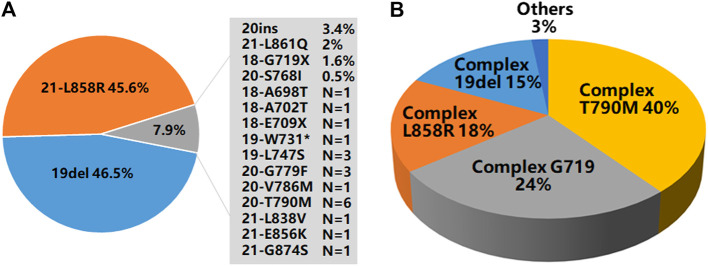
*EGFR* mutation patterns. **(A)** An overview of *EGFR* mutations present in 8,979 cases with single *EGFR* mutations. **(B)** A summary of *EGFR* mutations present in 642 cases with complex *EGFR* mutations.

Of the 9,621 cases with *EGFR* mutations, 93.3% of them (8,979/9,621) harbored a single *EGFR* mutation, including 8,274 (92.1%) cases carrying classical mutations (19del or L858R) and 705 (7.9%) cases carrying rare mutations while 6.7% of them (642/9,621) harbored complex *EGFR* mutations. Distribution of these *EGFR* mutations were shown in Figures 2A,B. The majority of the complex *EGFR* mutations occurred with coexistence of T790M (exon-20), G719X (exon-18), L858R (exon-21) and 19del (Figure 2B).

Among the *EGFR* mutations identified in the 9,621 cases, a total of 72 distinct variants could be recognized (different exon-19 deletions were classified as 19del, various exon-20 insertions were grouped as 20ins, and different G719, E709 and R776 mutations were labeled as G719X, E709X and R776X). NGS, Sanger sequencing and real-time PCR detected 76, 46, and 36% of the distinct *EGFR* mutations respectively. For the cases with single uncommon *EGFR* mutation, NGS detected 94% of them, while Sanger sequencing and real-time PCR detected 41% of them. For complex *EGFR* mutations, NGS detected 71% of them, while Sanger sequencing and real-time PCR detected 47% and 35% respectively. Details about these *EGFR* mutations were shown in Supplementary Tables S1 and S2. Of the 72 distinct *EGFR* variants, 15 of them (single or complex *EGFR* mutations) were newly identified in NSCLC (11 of them detected by NGS, 2 of them detected by Sanger sequencing and 3 detected by real-time PCR, with 1 detected by both Sanger sequencing and NGS). These newly identified variants were shown in Table 2.

**TABLE 2:** Novel *EGFR* mutations in NSCLC identified in this project.

*EGFR* mutations	Numbers of cases
A702T (exon 18)	1
E865K (exon 21)	1
G719X (exon 18) + L747V (exon 19)	2
G719X (exon 18) + 20ins (exon 20)	1
G719X (exon 18) + L833V (exon 21)	3
G719X (exon 18) + L858R (exon 21) + L861Q (exon 21)	1
19del (exon 19) + K728E (exon 18)	1
L858R (exon 21) + I706T (exon 18)	3
L858R (exon 21) + T790M (exon 20) + D761Y (exon 19)	1
L858R (exon 21) + R831H (exon 21)	1
L858R (exon 21) + A859S (exon 21)	1
L858R (exon 21) + L861Q (exon 21)	1
L858R (exon 21) + A871E (exon 21) + T790M (exon 20)	1
L861Q (exon 21) + G779C (exon 20)	1
L861Q (exon 21) + E865G (exon 21)	1

### Actionable Mutations in Non-Epidermal Growth Factor Receptor Genes Identified by Next-Generation Sequencing

**FIGURE 3:**
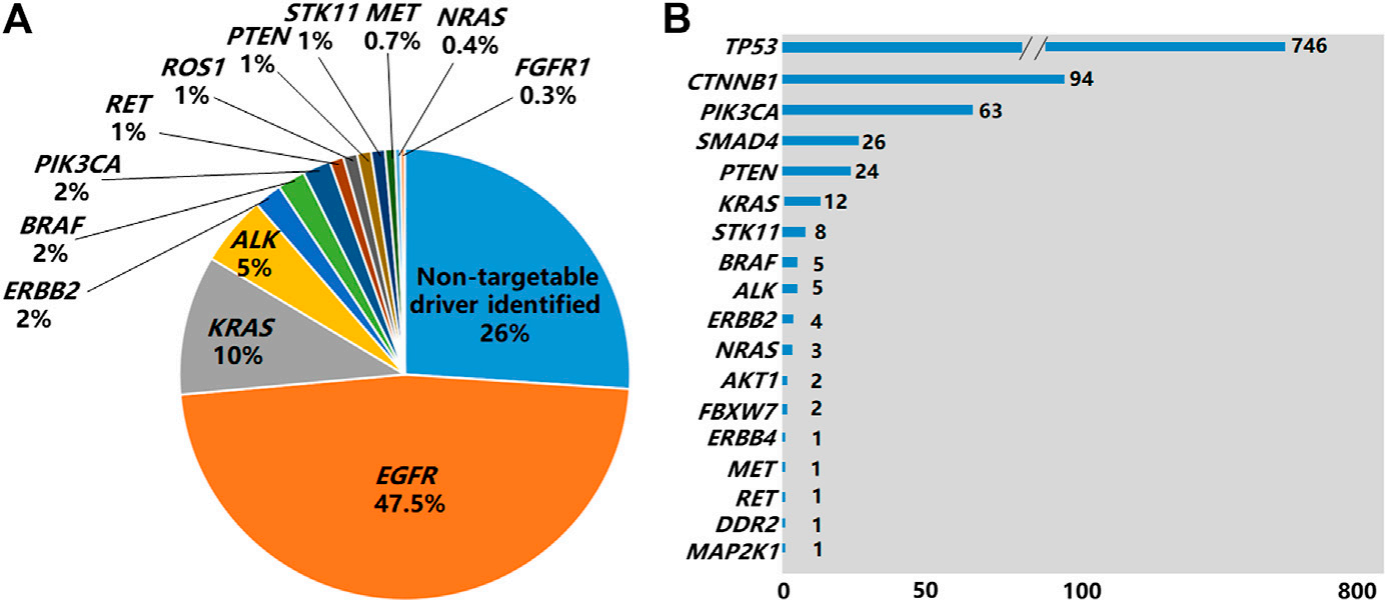
Non-*EGFR* genes carrying mutations identified by NGS. **(A)** Genes carrying targetable driver mutations identified in the 2,751 NSCLC cases by NGS. **(B)** Non-*EGFR* genes carrying mutations identified by NGS in 1,308 *EGFR*-positive cases by NGS.

Of the 2,751 cases tested by NGS, 52.5% of them (1,443/2,751) were found to be *EGFR* mutation negative. The 1,443 cases could be grouped into 3 subgroups: 1) 27% (*N* = 742,742/2,751) of them were found to have actionable mutations in the non-*EGFR* genes with available targeted drugs or potential drugs under clinical trials, including *ALK*, *ROS1*, *RET* fusions and other oncogenic events (Figure 3A) as well as *MAP2K1* (0.1%), *AKT1* (0.1%) and *FGFR3* (0.1%) (Data not shown in Figure 3A); 2) 18.3% (N = 504,504/2,751) were found to have tumor initiation- and/or progression-related mutations in non-*EGFR* genes, such as *TP53, SMAD4, FBXW7, CTNNB1* and *NOTCH1.* However there are no targeted drugs or potential drugs available to these mutated genes products currently; 3) 7.2% (*N* = 197,197/2,751) of them were found to carry neither oncogenic mutations in *EGFR* nor tumor initiation- and/or progression-related mutations in non-*EGFR* genes*.*

Of the 1,308 cases with *EGFR* mutations found by NGS, 65.3% of the cases (854/1,308) harbored non-*EGFR* mutations in 18 tumor-related genes, with *TP53* being the most frequently mutated (Figure 3B). The overall co-existing frequencies of *EGFR/KRAS*, *EGFR/ALK*, *EGFR/BRAF*, *EGFR/NRAS* were 0.4% (12/2,751), 0.2% (5/2,751), 0.2% (5/2,751) and 0.1% (3/2,751) respectively. *EGFR/KRAS* co-mutations were found in 0.9% (12/1,308) of the 1,308 patients with *EGFR* mutations and 4% (12/299) of the 299 patients with *KRAS* mutations. *EGFR/ALK* co-mutations were found in 3.8% (5/131) of the 131 patients with *ALK* fusions. *EGFR/BRAF* co-mutations were detected in 10.6% (5/47) of the 47 patients with *BRAF* mutations. *EGFR/NRAS* co-mutations were detected in 30% (3/10) of the 10 patients with *NRAS* mutations. In addition, we found 1 patient carrying both *EGFR* mutation and *RET* fusion. No patient was found to carry *EGFR* mutation co-existing with *ROS1* fusion or *Met* exon 14 skipping mutation in this cohort (Supplementary Table S4). Altogether, 83.8% (716/854) of patients carried mutations in 2 genes, 14.6% (125/854) of them carried mutations in 3 genes, and 1.5% (13/854) of them carried mutations in 4 genes (Supplementary Table S3).

We compared the mutation rates of some driver genes including *EGFR*, *ALK*, *ROS1*, *RET* and *KRAS* by the two different platforms of NGS testing. The positive detection rates of amplicon-based vs capture-based sequencing were 47.8% (521/1089) vs. 47.4% (787/1662) for *EGFR*, 5.7% (62/1089) vs. 4.2% (69/1662) for *ALK* fusions, 0.9% (10/1089) vs. 1% (17/1662) for *ROS1* fusions, 1.3% (14/1089) vs. 1.5% (25/1662) for *RET* fusions. Statistically, there were no significant difference about the detection rates for these driver gene mutations by the 2 NGS methods (*p* > 0.05), although amplicon-based method showed relatively higher detection rates for ALK fusions compared to capture-based method.

## Discussion

To our knowledge, this research project represents the largest data analysis of *EGFR* mutational status in Chinese patients with NSCLC by multiple platforms, providing several interesting findings valuable in clinical settings.

NGS testing expanded mutational spectrum of *EGFR* in NSCLC patients. Although only 2,751 of the 21,324 (12.9%) NSCLC specimens were tested by NGS, we identified 11 novel *EGFR* mutations (not being previously reported in NSCLC) in exons-18 to -21 by the NGS methods while Sanger sequencing and real-time PCR identified only 2 and 3 novel *EGFR* mutations in this region respectively (Supplementary Tables S1 and S2). We believe that if all of the 21,324 NSCLC specimens had been analyzed by NGS, all of the rare variants found by Sanger sequencing and real-time PCR would have been identified. Although both NGS and Sanger sequencing detected the region covering exons-18 to -21 of *EGFR*, Sanger sequencing will miss those *EGFR* mutations with frequencies of mutant alleles less than 20% (cut-off value determined in our validated data) due to its low technical sensitivity. Both NGS and real-time PCR showed similar technical sensitivity (1% frequency of mutant alleles), but real-time PCR method was designed to detect only 29 hotspot mutations of *EGFR* and might have missed mutations in the non-hotspot regions. Furthermore, real-time PCR couldn’t distinguish the differences within or around 19del, G719X, 20ins variants, for example G719A or G719C, though these variants may show different responses to EGFR-TKIs [18–20] (Table 3). In summary, NGS method shows obvious advantages over Sanger sequencing and real-time PCR methods for identifying novel actionable *EGFR* mutations.

**TABLE 3:** A summary of *EGFR* mutations tested by Sanger sequencing, real-time PCR and NGS platforms for NSCLC patients in this project.

Specimens	Sanger sequencing	Real-time PCR	NGS
Number of samples	5,244	13,329	2,751
*EGFR* mutation rate	40.3%	46.5%	47.5%
*EGFR* mutation types	46%	36%	76%
Covered regions of *EGFR*	18–21 exons	18–21 exons hotspots^a^	All coding sequencing^b^
Covered non*-EGFR*	No	No	Yes^c^
Technical sensitivity	20%	1%	1%
Recommended TCC	≥40%	≥1%	≥1%
Mean TAT (days)	5	4	8

^a^ 19del, G719X, 20ins subtypes could not be distinguished.

^b^ Capture-based NGS.

^c^ About 27% cases without *EGFR* mutations were identified by NGS to have non- *EGFR* driver events with available targeted drugs or potential drugs. TCC: tumor cell content.

NGS identified a long list of non-*EGFR* mutations related to tumor initiation and progression, adding additional therapeutic opportunities and/or assessing the prognostic outcomes for NSCLC patients. In this study, 52.5% (1,443/2,751) of the cases tested by NGS did not carry *EGFR* mutations, similar to the results reported in previous Asian lung adenocarcinoma series [10]. Of the cases tested by NGS, 27% of them were found to have actionable mutations, including *ALK* (5%), *ROS1* (1%), *BRAF* (2%), *KRAS* (10%), *RET* (1%), *ERBB2* (*HER2*) (2%), and exon-14 skipping mutations of *MET* (0.7%) as well as other genes, such as *PIK3CA* (2%), *PTEN* (1%), *STK11* (1%) *NRAS* (<0.5%), *FGFR1* (<0.5%), *FGFR3* (<0.5%), *MAP2K1* (<0.5%), and *AKT1* (<0.5%). For *ALK*, *ROS1* and *BRAF* V600E alterations, targeted drugs have been available in clinical setting. For the other genes in this list, potential targeted drugs providing possible therapeutic opportunities for NSCLC patients are emerging in some international clinical trials (www.clinicaltrials.gov). In addition, 18.3% of the cases tested by NGS carried tumor-mutations in non-*EGFR* genes classified currently as non-actionable, such as *TP53, SMAD4, FBXW7, CTNNB1* and *NOTCH1.* However, pathogenic variants in these genes were considered to have prognostic or predictive significances for NSCLC patients [21, 22]. For the 7.2% of the cases tested by NGS without finding any of tier 1 and tier 2 mutations in neither *EGFR* nor non*-EGFR* genes, but we think tier 3 variants (variants of unknown clinical significance-VUS, not reported) found in these cases might represent potentially useful biomarkers for monitoring cancer treatment effects *via* liquid biopsy. Interestingly, of the 1,308 cases with *EGFR* mutations found by NGS, 65.3% of the cases (854/1,308) also harbored non-*EGFR* mutations in 18 tumor-related genes. In NSCLC, oncogenic driver mutations are typically mutually exclusive. However, cases of mutations in multiple driver genes are increasingly reported, as well as in our retrospective study. *EGFR* and other driver genes including *KRAS*, *ALK*, *BRAF*, *NRAS*, *RET* co-alterations are likely to represent certain proportion of cases with multiple mutations in NSCLC. These co-alterations may provide prognostic or predictive effects to EGFR-TKIs as reported previously [23–26].

NGS is the method which could maximize the findings of *EGFR* mutations as shown in this project. The total *EGFR* positive mutation rate in our data was 45.1%, concordant with several previous studies of Chinese patients with NSCLC [10, 16]. By comparing the *EGFR* mutation detection rates showed by the 3 methods, 40.3% (2,111/5,244) by Sanger sequencing was significantly lower than that found by real-time PCR (46.5%, 6,202/13,329) and NGS (47.5%, 1,308/2,751) (*p* < 0.001), indicating that the Sanger sequencing had missed some *EGFR* mutations which would have been identified if NGS or real-time PCR methods had been applied. The main reason leading to higher detection rates by NGS or real-time PCR is attributed to higher sensitivities of the NGS and real-time PCR (1% frequency of mutant alleles) than that of Sanger sequencing (20% frequency of mutant alleles) (Table 3). As expected, comparable *EGFR*-positive rates identified by real-time PCR and NGS were observed in the current study. By stratifying different levels of TCC from 1 to 20% in tumor specimens for analyzing *EGFR* detection rates, there were no significant differences for *EGFR* positive mutations rates by either real-time PCR or NGS. This finding suggested that *EGFR* mutations present in these specimens with TCC from 1 to 20% could be fairly identified by real-time PCR or NGS, providing therapeutic opportunities using EGFR-TKIs for these patients. *EGFR* mutation rates by Sanger sequencing in the FFPE samples with <40 and ≥40% TCC were 30.8% (757/2,454) and 48.5% (1,354/2790) respectively, showing similar *EGFR* positive rates in FFPE samples with TCC ≥40% by the 3 methods. Based on these findings, we recommend *EGFR* mutation testing of specimens with ≥1% TCC by NGS or real-time PCR. Sanger sequencing could be considered for specimens with ≥40% TCC due to its low cost (Table 3). However, considering the facts that the *EGFR* VAFs overlapped greatly among groups with different TCC, showing high levels of variations regarding *EGFR* VAFs ranging from 0.01 to 0.97 (EGFR VAF was up to 0.675 in one sample with 1% TCC, down to 0.01 in another sample with 20% TCC), some low level of *EGFR* VAFs in high level TCC specimens could have been missed by Sanger sequencing method.

We acknowledge that there are several limitations or concerns in this research: 1) Since some features, such as pathologic diagnosis (adenocarcinoma, squamous or other histological types), smoking status, grades, and stages of the disease, were not fully described in their requisition forms when *EGFR* testing were ordered for therapeutic purpose, further stratification analysis were not implemented. 2) Follow-up data were not available for monitoring responses of TKIs treatment targeting to those uncommon *EGFR* mutations, complex *EGFR* mutations or co-existing mutations in both *EGFR* and non-*EGFR* genes. Even for NSCLC patients with a classical *EGFR* mutation (L858R in exon-21 or 19del), it was speculated that different outcomes after EGFR-TKIs treatments might be present [27]. 3) Although some identified novel variants of *EGFR* and non-*EGFR* were considered relevant to the initiation and progression of NSCLC, further investigations about their abnormal functions and pathogenicity are required.

In conclusions, we presented a large dataset of *EGFR* mutations in Chinese NSCLC patients including a long list of novel *EGFR* mutations identified, providing valuable information for the diagnosis and subsequent treatment of the disease. NGS method showed advantages over Sanger sequencing and real-time PCR not only by providing the highest mutation detection rates of *EGFR* but also by identifying actionable non-*EGFR* mutations with available targeted drugs in clinical setting.

## Data Availability

Data are available from the corresponding author on reasonable request.
