# Supplementary material for: Mutation Spectrum of EGFR From 21,324 Chinese Patients With Non-Small Cell Lung Cancer (NSCLC) Successfully Tested by Multiple Methods in a CAP-Accredited Laboratory
Source: Pathol Oncol Res. 2021 Apr 7;27:602726. doi: 10.3389/pore.2021.602726 (PMC8262202; doi:10.3389/pore.2021.602726)
Supplement: Supplementary file 1 [file DataSheet1.PDF]

Mutation Spectrum of *EGFR* from 21,324 Chinese Patients with Non-Small Cell Lung Cancer (NSCLC) Successfully Tested by Multiple Methods in a CAP-Accredited Laboratory

Linlin Mao[1] . Weiwei Zhao[1] . Xiaoxia Li[1] . Shangfei Zhang[1] . Changhong Zhou[1] . Danyan Zhou[1] . Xiaohua Ou[1,2] . Yanyan Xu[1] . Yuanxiao Tang[1] . Xiaoyong Ou[1] . Changming Hu[1] . Xiangdong Ding[3] . Pifu Luo[3] . Shihui Yu[1,2]

[1]Clinical Genome Center, KingMed Diagnostics, Guangzhou, China

[2]Institute of KingMed Translational Medicine, Guangzhou, China

[3]Department of Pathology, KingMed Diagnostics, Guangzhou, China

Linlin Mao and Weiwei Zhao contributed equally to the manuscript.

Corresponding author: Dr. Shihui Yu, KingMed Diagnostics, Guangzhou, 510320, China

Tel: +86-13825052332; Email: zb-yushihui@kingmed.com.cn (S., Yu)

Journal Name：Pathology & Oncology Research

Supplementary Materials:

Genotyping *EGFR* by Sanger Sequencing

Sequence variants in the *EGFR* exons 18-21 were scanned by Sanger sequencing using the primers listed in the table below. Nested PCR was applied in the detection. The reaction mixture was prepared in a final volume of 25 µL as follows: Nest A: 12.5 µL Q5® High-Fidelity 2X Master Mix (NEB #M0515), 8.5 µL ddH_2O, 1.0 µL Forward primer, 1.0 µL Reverse primer, and 2 µL DNA Sample. Nest B: 12.5 µL Q5® High-Fidelity 2X Master Mix (NEB #M0515), 8.5 µL ddH_2O, 1.0 µL Forward primer, 1.0 µL Reverse primer, and 2 µL Nest A PCR products.

The PCR cycling and melting conditions were as follows: Nest A: an initial incubation at 98ºC for 30 s, followed by 30 cycles of 98ºC for 10 s, 58ºC for 30 s, and 72ºC for 1 min; final extension step at 72ºC for 5 min. Nest B: an initial incubation at 98ºC for 30 s, followed by 35 cycles of 98ºC for 10 s, 62ºC for 30 s, and 72ºC for 1 min; final extension step at 72ºC for 5 min.

The Nest B PCR products were sequenced by Sanger sequencing.

Table: Primers for genotyping *EGFR* by Sanger sequencing

Primer Set	Size (bp)	Primer Name	Primer Sequence
EGFR-EXON18	246	EGFR-X18A-Fwd	GAGGTGACCCTTGTCTCTGTGT
		EGFR-X18A-Rev	TATACAGCTTGCAAGGACTCTGG
	213	EGFR-X18B-M13F	tgtaaaacgacggccagtCCTTGTCTCTGTGTTCTTGTCC
		EGFR-X18B-M13R	caggaaacagctatgaccTCCCCACCAGACCATGAGAG
EGFR-EXON19	244	EGFR-X19A-Fwd	GCTGGTAACATCCACCCAGA
		EGFR-X19A-Rev	AAAAGGTGGGCCTGAGGTTCA
	184	EGFR-X19B-M13F	tgtaaaacgacggccagtACAATTGCCAGTTAACGTCTTCC
		EGFR-X19B-M13R	caggaaacagctatgaccGAGGTTCAGAGCCATGGACC
EGFR-EXON20	326	EGFR-X20A-Fwd	CCACCATGCGAAGCCACACT

		EGFR-X20A-Rev	ATCCCCATGGCAAACTCTTG
	261	EGFR-X20B-M13F	tgtaaaacgacggccagtCCACCATGCGAAGCCACACT
	261	EGFR-X20B-M13R	caggaaacagctatgaccCTCCCCTCCCCGTATCTCCCT
EGFR-EXON21	254	EGFR-X21A-Fwd	GAGCTTCTTCCCATGATGATCT
EGFR-EXON21	254	EGFR-X21A-Rev	CCTGGTGTCAGGAAAATGCT
EGFR-EXON21	224	EGFR-X21B-M13F	tgtaaaacgacggccagtTGATCTGTCCCTCACAGCAG
EGFR-EXON21	224	EGFR-X21B-M13R	caggaaacagctatgaccAATGCTGGCTGACCTAAAGC
sequencing primer		M13F	tgtaaaacgacggccagt
sequencing primer		M13R	caggaaacagctatgacc

Genotyping *EGFR* by Real-Time PCR

The therascreen EGFR RGQ PCR Kit (Qiagen China, Shanghai, China) was used to detect 29 mutation hotspots in the *EGFR* exons 18-21. The 29 mutation hotspots are as following: 19 deletions in exon 19 (detecting the presence of any of 19 deletions but cannot distinguish them); T790M; L858R; L861Q; G719X (cannot distinguish G719S, G719A, or G719C.); S768I; 3 insertions in exon 20 (can detect the presence of any of the 3 insertions, cannot distinguish them).

The assay was carried out according to the manufacturer’s protocol with the Rotor-Gene Q instrument. The 25 µL RT-PCR reaction system consisted of 19.5 µL Control Reaction Mix (Ctrl), 0.5 µL Taq DNA polymerase (Taq), and 5 µL of sample DNA. PCR was performed with initial denaturation at 95ºC for 15min, followed by 40 cycles of amplification (at 95ºC for 30s and 61ºC for 1min). The results were analyzed according to the criteria defined by the manufacturer’s instructions.

NGS Analysis

For the first 1,089 non-small-cell lung cancer (NSCLC) cases, the Ion AmpliSeq™ Library Kit 2.0 (ThermoFisher, Waltham, USA) was used to prepare the libraries. The targeted regions were amplified by combining Ion Ampliseq HiFi Master Mix and Ion AmpliSeq™ Colon and Lung Cancer Panel V2 and Ion AmpliSeq™ RNA Fusion panel which cover point mutations and small insertions and deletions (indels) of 22 genes (*EGFR*, *ALK*, *BRAF*, *KRAS*, *MET*, *ERBB2*, *AKT1*, *CTNNB1*, *ERBB4*, *DDR2*, *FBXW7*, *FGFR1*, *FGFR2*, *FGFR3*, *MAP2K1*, *NOTCH1*, *NRAS*, *PIK3CA*, *PTEN*, *SMAD4*, *STK11 and TP53*) as well as fusions of 4 genes (*ALK*, *ROS1*, *RET*, *NTRK1*). The PCR conditions were as follows: 99°C for 2 min, 20 cycles of 99°C for 15 seconds and 60°C for 4 min, with a final hold at 10°C until use. Then, amplicons were then treated with FuPa reagent to perform a partial digestion step with the following conditions: 50°C for 10 min, 55°C for 10 min, 60°C for 20 min, with a final hold at 10°C until use. Barcode adapters were added to the amplicons, and the reaction products were purified with AMPure XP beads (Beckman Coulter, Brea, USA). The final ligated products were eluted and amplified via the following PCR conditions: 98°C for 2 min, 5 cycles of 98°C for 15 s and 64°C for 1 min, with a final hold at 10°C until use. After the purification step, the concentrations of the libraries were estimated by Qubit 3.0 Fluorometer. The library templates were prepared using the Ion PGM™ Hi-Q™ OT2 Kit (ThermoFisher, Waltham, USA). The products were then sequenced on an Ion PGM instrument (ThermoFisher, Waltham, USA) using the Ion PGM Hi-Q Sequencing Kit (ThermoFisher, Waltham, USA) according to the manufacturer’s instructions.

For the remaining 1,662 NSCLC cases, we adopted a validated capture-based method. Details were as follows: genomic libraries were prepared using the KAPA HyperPlus Kit (Kapa Biosystems, Wilmington, USA) with an initial amount of 10 to 50 ng of DNA. The tissue DNA was fragmented with Frag Buffer and Frag enzyme for 20 min at 37°C. The fragmented tissue DNA and the extracted cfDNA were incubated with end repair and A-tailing reagents for 30 min at 65°C. Next, specific index adapters, ligation buffer, DNA ligase and PCR-grade water were added to the mixture followed by incubation at 20°C for 20 min. The ligated products were then purified with AMPure XP beads (Beckman Coulter, Brea, USA). The amplification steps were performed using KAPA HiFi HotStart ReadyMix and a library amplification primer mix with the following conditions: 98°C for 40 seconds, 8 cycles of 98°C for 15 seconds and 60°C for 30 seconds, 72°C for 30 seconds, 10°C for 1 min, with a final hold at 10°C until use. Hybridization and wash kits and custom-designed probes (xGen Lockdown Reagents and custom panel for 83 genes covering the genes and targeted regions listed above, Integrated DNA Technologies, Inc., Coralville, USA) were used to the perform hybrid capturing. The tissue DNA libraries or cfDNA libraries were bound to the biotin-labeled probes and hybridized for more than 14 hours at 65°C, followed by elution using xGen wash buffer solution according to manufacturer's guidelines (Integrated DNA Technologies, Inc., Coralville, USA). The NGS libraries were sequenced using Illumina Nextseq 500 or NovaSeq 6000 instruments (Illumina, San Diego, USA).

Supplementary Table 1 Cases carrying single *EGFR* mutations identified by multiple platforms

Single EGFR mutation	Cases detected by different platforms			Reported in NSCLC
	Sanger	Real-time PCR	NGS	
A698T (exon 18)	/	/	1	Yes
A702T (exon 18)	/	/	1	No
E709X (exon 18)	/	/	1	Yes
G719X (exon 18)	19	118	8	Yes
19del (exon 19)	908	2717	551	Yes
W731* (exon 19)	/	/	1	Yes
L747S (exon 19)	/	/	3	Yes
20ins (exon 20)	100	144	61	Yes
S768I (exon 20)	20	27	1	Yes
G779F (exon 20)	2	/	1	Yes
V786M (exon 20)	/	/	1	Yes
T790M (exon 20)	/	6	/	Yes
L838V (exon 21)	/	/	1	Yes
L858R (exon 21)	832	2761	505	Yes
L861Q (exon 21)	50	110	27	Yes
E865K (exon 21)	/	/	1	No
G874S (exon 21)	/	/	1	Yes
Total	1931	5883	1165	

Supplementary Table 2 Cases carrying complex *EGFR* mutations identified by multiple platforms

Complex EGFR mutations	Cases detected by different platforms			Reported in NSCLC
	Sanger	Real-time PCR	NGS	
G719X (exon 18) + E709X (exon 18)	16	/	9	Yes
L858R (exon 21) + E709X (exon 18)	12	/	5	Yes
G719X (exon 18) + S720F (exon 18)	/	/	2	Yes
19del (exon 19) + G719X (exon 18)	6	6	/	Yes
G719X (exon 18) + L747V (exon 19)	/	/	2	No
G719X (exon 18) + 20ins (exon 20)	/	1	/	No
G719X (exon 18) + S768I (exon 20)	3	51	10	Yes
G719X (exon 18) + S768I (exon 20) + L858R (exon 21)	5	4	/	Yes
G719X (exon 18) + T790M (exon 20)	/	3	1	Yes
G719X (exon 18) + L833V (exon 21)	3	/	/	No
G719X (exon 18) + L858R (exon 21)	4	6	1	Yes
G719X (exon 18) + L861Q (exon 21)	10	26	10	Yes
G719X (exon 18) + L858R (exon 21) + L861Q (exon 21)	/	1	/	No
19del (exon 19) + K728E (exon 18)	/	/	1	No
19del (exon 19) + K754E (exon 19)	/	/	1	Yes
19del (exon 19) + 20ins (exon 20)	/	2	1	Yes
19del (exon 19) + S768I (exon 20)	2	/	/	Yes
19del (exon 19) + V769M (exon 20)	/	/	1	Yes
19del (exon 19) + T790M (exon 20)	7	56	25	Yes
19del (exon 19) + L858R (exon 21)	4	67	2	Yes
19del (exon 19) + T790M (exon 20) + C797S (exon 20)	/	/	1	Yes
19del (exon 19) + T790M (exon 20) + L858R (exon 21)	/	2	/	Yes
19del (exon 19) + L861Q (exon 21)	/	5	/	Yes
20ins (exon 20) + L858R (exon 21)	/	1	/	Yes
H773L (exon 20) + V774M (exon 20)	3	/	1	Yes
S768I (exon 20) + V769L (exon 20)	1	/	/	Yes
S768I (exon 20) + V774M (exon 20)	3	/	/	Yes
S768I (exon 20) + L858R (exon 21)	2	13	4	Yes
S768I (exon 20) + T790M (exon 20) + L858R (exon 21)	2	1	/	Yes
L858R (exon 21) + I706T (exon 18)	2	/	1	No
L858R (exon 21) + E709X (exon 18)	6	/	/	Yes
L858R (exon 21) + L747S (exon 19)	/	/	2	Yes

L858R (exon 21) + K757N (exon 19)	1	/	1	Yes
L858R (exon 21) + D761Y (exon 19)	/	/	3	Yes
L858R (exon 21) + R776X (exon 20)	12	/	7	Yes
L858R (exon 21) + T790M (exon 20)	60	71	26	Yes
L858R (exon 21) + T790M (exon 20) + D761Y (exon 19)	/	/	1	No
L858R (exon 21) + T790M (exon 20) + C797S (exon 20)	/	/	1	Yes
L858R (exon 21) + T790M (exon 20) + L792H (exon 20)	/	/	1	Yes
L858R (exon 21) + R831H (exon 21)	/	/	1	No
L858R (exon 21) + L833V (exon 21)	4	/	4	Yes
L858R (exon 21) + V834L (exon 21)	8	/	2	Yes
L858R (exon 21) + L838V (exon 21)	1	/	/	Yes
L858R (exon 21) + A859S (exon 21)	/	/	1	No
L858R (exon 21) + K860I (exon 21)	2	/	4	Yes
L858R (exon 21) + L861Q (exon 21)	/	1	/	No
L858R (exon 21) + A871E (exon 21) + T790M (exon 20)	/	/	1	No
L858R (exon 21) + A871G (exon 21)	/	/	2	Yes
L858R (exon 21) + G873E (exon 21)	/	/	1	Yes
S768I (exon 20) + V769L (exon 20)	/	/	2	Yes
S768I (exon 20) + V774M (exon 20)	/	/	1	Yes
L861Q (exon 21) + R776X (exon 20)	1	/	2	Yes
L861Q (exon 21) + G779C (exon 20)	/	/	1	No
L861Q (exon 21) + T790M (exon 20)	/	2	/	Yes
L861Q (exon 21) + E865G (exon 21)	/	/	1	No
Total	180	319	143	

Supplementary Table 3 Mutations in the non-*EGFR* genes co-existing with positive *EGFR* mutation tested by NGS

Mutations in the non-*EGFR* genes co-existing with positive *EGFR* mutation	Cases identified by NGS
EGFR,AKT1,TP53	1
EGFR,ALK fusion,TP53	5
EGFR,BRAF	2
EGFR,BRAF,AKT1	1
EGFR,BRAF,TP53	2
EGFR,CTNNB,SMAD4	5
EGFR,CTNNB1	43
EGFR,CTNNB1,PIK3CA	7
EGFR,CTNNB1,PIK3CA,TP53	2
EGFR,CTNNB1,PTEN,TP53	2
EGFR,CTNNB1,SMAD4,TP53	4
EGFR,CTNNB1,TP53	36
EGFR,DDR2,TP53	1
EGFR,ERBB2(HER2)	1
EGFR,ERBB2(HER2),STK11	1
EGFR,ERBB2(HER2),TP53	2
EGFR,ERBB4	1
EGFR,FBXW7,TP53	1
EGFR,FGFR3,TP53	1
EGFR,KRAS	4
EGFR,KRAS,FBXW7,PIK3CA	1
EGFR,KRAS,PIK3CA,TP53	1
EGFR,KRAS,PTEN	1
EGFR,KRAS,SMAD4,TP53	1
EGFR,KRAS,STK11	1
EGFR,KRAS,TP53	3
EGFR,MAP2K1(MEK1),PTEN,TP53	1
EGFR,MET(N375K)	1
EGFR,NRAS	2
EGFR,NRAS,TP53	1
EGFR,PIK3CA	21
EGFR,PIK3CA,PTEN,TP53	1
EGFR,PIK3CA,TP53	30
EGFR,PTEN	5
EGFR,PTEN,TP53	14
EGFR,RET fusion	1
EGFR,SMAD4	8

EGFR,SMAD4,TP53	8
EGFR,STK11	2
EGFR,STK11,TP53	4
EGFR,TP53	625
Total	854

Supplementary Table 4 *EGFR* and other important driver genes co-alterations tested by NGS were shown in details.

EGFR mutations	Co-occurring alterations in other important driver genes
EGFR L747_S752del (exon 19)	*KRAS* G12V
EGFR G719A (exon18) + S768I (exon 20)	*KRAS* G12D
EGFR L747_A750delinsP (exon 19)	*KRAS* Q61H
EGFR L858R (exon 21)	*KRAS* G12C
EGFR A702T (exon18)	*KRAS* G12D
EGFR L858R (exon 21)	*KRAS* G12A
EGFR L858R (exon 21) + R776C (exon 20)	*KRAS* G13D
EGFR L858R (exon 21)	*KRAS* G12V
EGFR E746_A750del (exon 19)	*KRAS* G12D
EGFR E746_A750del (exon 19)	*KRAS* G12V
EGFR E746_A750delinsQP (exon 19)	*KRAS* G13D
EGFR L858R (exon 21)	*KRAS* G12A
EGFR E746_A750del (exon 19)	*EML4/ALK* fusion
EGFR L858R (exon 21) + K860I (exon 21)	*EML4/ALK* fusion
EGFR L858R (exon 21)	*EML4/ALK* fusion
EGFR L858R (exon 21)	*EML4/ALK* fusion
EGFR L858R (exon 21)	*KIF5B/ALK* fusion
EGFR E746_A750del (exon 19) + T790M (exon 20)	*BRAF* V600E
EGFR L858R (exon 21)	*BRAF* V600E
EGFR L858R (exon 21)	*BRAF* V600E
EGFR G719D (exon18)	*BRAF* V600E
EGFR E746_A750del (exon 19)	*BRAF* K601E
EGFR L858R (exon 21)	*NRAS* G13C
EGFR E746_A750del (exon 19)	*NRAS* G12A
EGFR L858R (exon 21)	*NRAS* G12V
EGFR E746_A750del (exon 19)	*KIF5B/RET* fusion

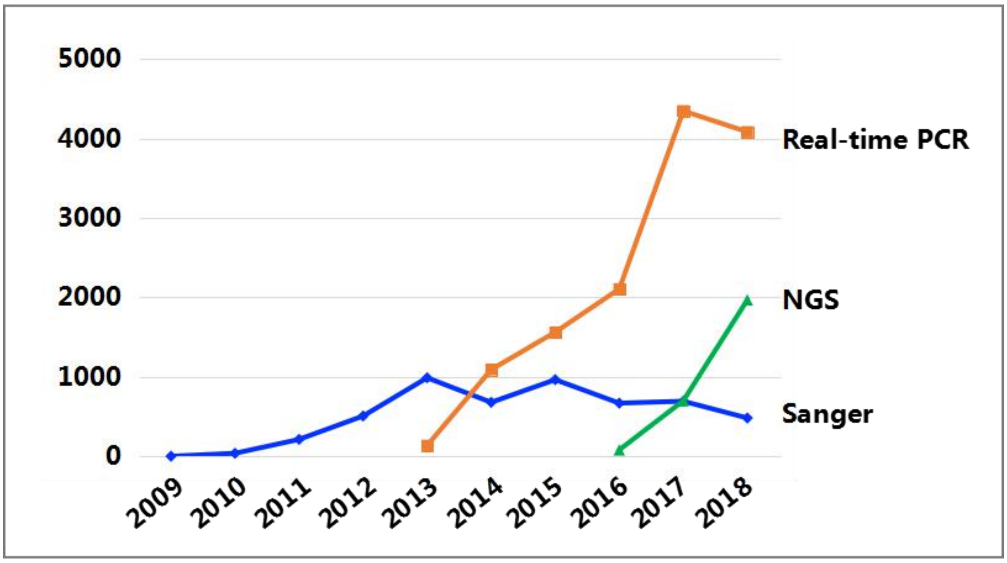

Supplementary Fig. 1 The numbers of cases tested by different methods (Sanger sequencing, real-time PCR, and NGS). These methods started to be implemented in our laboratory from 2009, 2013 and 2016 respectively.
